# Mythical compounds

**DOI:** 10.1107/S205322962300791X

**Published:** 2023-10-24

**Authors:** Phillip E. Fanwick

**Affiliations:** a Department of Chemistry, Purdue University, West Lafayette, IN 47907, USA

**Keywords:** crystallographic errors, atom misassignments, incorrect modeling, disordered guest mol­ecules, incorrect space group, incorrect cell size, checkCIF

## Abstract

There is a need for validation methods to ensure the quality and consistency of reported data, but a recent article by Raymond and Girolami [*Acta Cryst.* (2023), C**79**, https://doi.org/10.1107/S2053229623007088] will alert chemists, crystallographers, referees, and editors to not always trust the crystal structure but also to ensure that the chemistry is consistent with previous chemistry.

As crystallography became readily available, it was obvious there was a need for validation methods to ensure the quality and consistency of the reported data. While there were several publications indicating this need, the publication that really provided the impetus was entitled *Some Incorrect Space Groups* in Volume 16 of *Inorganic Chemistry* by Richard Marsh and Verner Schomaker (Marsh & Schomaker, 1979 ▸). Dick Marsh con­tinued publishing similar lists of incorrect space groups for many years and in fact being cited in such a publication was referred to as ‘being Marshed’. The only problem discussed in these publications was the choice of space group, though some required a change in the Laue class. If something so basic to crystallography as the space group was not being determined correctly, one could only imagine the extent of other problems. Collecting crystallographic data, structure solution, and structure refinement required the crystallographer to make many decisions. Frequently, the researcher will be biased towards a desired result and may force the structure to be consistent with the expected model. It was obvious some program was needed to check results.

However, before validation programs could be written, a universal formatted output from crystallographic experiments was needed. The program would provide a means to report all the crystallographic data, as well as the metadata, which could help in validating the methods used and ensure all the required data were provided. This required output was the crystallographic information file (CIF) format (Hall *et al.*, 1991 ▸).

With the development of CIF, it was now possible to create software that could carry out validation of crystal structures. The most widely used such software is *checkCIF* by Ton Spek (Spek, 2020 ▸). This program was first reported in 2003 (Spek, 2003 ▸) and has found widespread use ever since. The program provided an extensive validation of submitted data and found widespread adoption. Once instituted, many complained of the problems of generating CIF files and understanding and correcting the errors *checkCIF* reported. However, both CIF and *checkCIF* have proved vital to the growth of good crystallography. Many reports of questionable or incorrect crystallography have been caught before they entered the literature and they alerted many producing and publishing crys­tallographic results of potential problems.

The use of crystal structure validation has led to the assumption that validation to ensure that the crystallographic results are correct means the chemical results must also be correct. An article by Kenneth Raymond and Gregory Girolami (Raymond & Girolami, 2023 ▸) proves that this assumption is incorrect. This article reviews published reports that all contain inaccurate chemical conclusions. These structures were published in science journals which are all peer reviewed. The erroneous structures were published from 1976 to 2019, so, in most cases, current software and diffractometers were used. A majority were published after 1999. Not surprisingly, many of these structures were offered as reports of new or highly unusual compounds which later proved to be non-existent.

Raymond and Girolami note several classes of chemically incorrect structures. The first class is the obvious case of structures with incorrectly assigned atoms. The second is high-symmetry superstructures which include guest mol­ecules. The third class is cases of an incorrectly assigned space group which also results in incorrect crystallography. The last case is that of incorrectly determined unit cells, again a crystallographic error.

Included with all the incorrect results presented by Raymond and Girolami are brief discussions of why the results are wrong and explanations or, in some cases, speculation as to how they ended up with incorrect chemistry. The most chemically incorrect structure is a report of a series of new divalent lanthanide compounds Ni_2_[LnCl_6_] (Ln = Eu, Ce, and Gd) (Baldo *et al.*, 2018 ▸). All turned out to be the com­plex [Ni(NH_3_)_6_]^2+^ with a disordered anion of chloride and nitrate. By coincidence, the three elements Ln, Ni, and Cl all have atomic numbers about 2.3 times greater than the actual elements of Ni, a mixture of Cl and N, and N. Since the ratio of the atomic numbers was nearly the same in both the correct and incorrect formulation, the structures refined because the scale factor was able to adjust for the incorrect number of electrons.

It is clear that just validating the crystallography is insufficient to ensure the correctness of the formula, connectivity, and geometric parameters from structural determinations. Hopefully this article by Raymond and Girolami will alert and alarm chemists, crystallographers, referees, and editors of the need to not always trust the crystal structure but also to ensure that the chemistry is consistent with previous chemistry. This is especially true for reports of new or totally unexpected com­pounds. Maybe it will encourage some in the crystallographic community (if it is not already being done) to try to apply the advances in artificial intelligence using the copious amount of data in the various crystallographic databases to create a chemistry validation program for crystal structures. David Watkin of Oxford University has frequently labeled crystallography as the ‘Gold Standard’ of analytical methods. More must be done to ensure in the future it will not lose its luster.<!?tpb=5.6pt>
